# A case study showing the role of hydrophobicity variants and other enriched mAb proteoforms on filterability through a virus filter with productivity improvement measures

**DOI:** 10.1002/btpr.70101

**Published:** 2026-01-19

**Authors:** Solomon Isu, Derek Silva, Melissa Holstein, Angela Lewandowski, Kristina Cunningham, Adam Sokolnicki, Bala Raghunath

**Affiliations:** ^1^ Technical and Scientific Solutions MilliporeSigma Burlington Massachusetts USA; ^2^ Bioanalytical Research and Development MilliporeSigma Bedford Massachusetts USA; ^3^ Biologics Development Bristol Myers Squibb Devens Massachusetts USA

**Keywords:** chromatography, fractionation, mAbs, proteoforms, relative hydrophobicity variants

## Abstract

A rapid assessment of manufacturability for drug candidates is crucial for advancing a prospective biotherapeutic from a candidate to a bulk drug substance. A lot‐to‐lot approach to manufacturability is adopted where each biologic batch is assessed for manufacturability as a bulk, unfractionated pool. Manufacturers may explore a more granular approach, independently enriching and evaluating the filterability of antibody variants within each lot, especially within the confines of relative hydrophobicity and surface charge. This study examined the use of bind‐and‐elute chromatography to alter the proportions of monoclonal antibody (mAb) proteoforms in eluate sub‐pools from a mixed‐mode chromatography resin‐packed column. Filterability of each sub‐pool through a virus‐retaining filter was subsequently examined. Circular dichroism and Fourier transform infrared spectroscopy were performed for each sub‐pool to probe for higher‐order structure differences between mAb variants enriched therein. Bioanalytical techniques were also used to assess colloidal stability, surface hydrophobicity, surface charge, and size differences. Results showed that basic charge variants, high‐mannose glycovariants, high relative hydrophobicity proteoforms, and high‐molecular‐weight species were enriched in the last‐eluting (terminal) sub‐pools. The first sub‐pool and the final sub‐pool showed the most fouling propensity on VPro virus filters. Circular dichroism showed that enriched proteoforms in the last sub‐pool possessed a higher percentage of bends. Most secondary structures did not vary significantly between sub‐pools. Diffusion interaction parameter was highly negative across all sub‐pools and the bulk unfractionated pool. These results provide a design space for identifying and depleting problematic mAb variants before the crucial virus filtration step.

## 1 INTRODUCTION

Monoclonal antibodies (mAbs) constitute a considerable proportion of the biopharmaceutical industry. Global revenue from antibody sales amounted to US$20 billion nearly two decades ago.^1^ Six different antibody drugs posted revenues exceeding US$9 billion in 2023, and there are currently more than 20 blockbuster mAbs on the market.^2^ Global revenue from antibody‐based drugs is projected to exceed US$300 billion in 2025.^3^, ^4^ Antibodies have applications in oncology, autoimmune and inflammatory disorders, genetic diseases, cardiovascular diseases, and macular degeneration, among others.^3^, ^5^

Manufacturability assessment of mAbs helps identify drug candidates that would be challenging for the chemistry, manufacturing, and controls (CMC) team to develop practical chromatography and virus filtration platforms. Virus filtration (VF) is a regulatory requirement for cell culture‐derived biologics. VF is a size‐based (20‐nm pore size) filtration unit operation that excludes viruses and other larger impurities while allowing the biologic (typically 5–12 nm and less than 170 kDa) to pass through in normal‐flow mode.^6^ The VF step is considered a rate‐determining unit operation in downstream purification because the therapeutic protein or mAb typically fouls the VF membrane through multiple mechanisms, including mechanical blocking and adsorptive caking.^7^ Simultaneously, the VF step is perceived as a purification bottleneck because of the minimal size disparity between the desired drug molecule and any adventitious virus impurity such as parvoviruses or adeno associated viruses that are about 20–25 nm in size.^8^, ^9^ The VF membrane acts as a final checkpoint for any viral particles and is designed to ensure 99.99 percent removal of enveloped and non‐enveloped viruses while ensuring high recovery of the drug substance batch.^10^ Rapid filtration of the drug substance with minimal membrane fouling is a desirable attribute of VF membranes to minimize product deterioration due to batch reprocessing or loss of an entire batch due to prematurely clogged membranes.^7^, ^11^, ^12^

Traditionally, the manufacturability of mAbs has been assessed normatively across entire harvest pools, utilizing specific cell lines and bioreactor conditions. While it is pertinent to study the fouling mechanisms of entire mAb pools on VF membranes on a lot‐to‐lot basis, a more granular approach may be required to evaluate the contribution of different mAb proteoforms (within a batch) to membrane fouling. These microheterogenous variants are usually present at various ratios in a production lot or batch and constitute the overall measurable quality attribute. Such quality attributes that may differ within proteoforms include surface charge, surface hydrophobicity, hydrodynamic diameter, colloidal stability, and glycan types. Manufacturers may utilize unit operations before VF (e.g., chromatography polishing) to enrich or deplete different mAb variant groups to identify VF membrane fouling culprits.

The concept of hydrophobicity variants is well established and is governed by the higher‐order structure of mAb proteoforms, in which the exposure or concealment of hydrophobic residues within antibody polypeptide chains can lead to differences in surface hydrophobicity.^13^, ^14^, ^15^ Bolton et al.^16^ reported that the component of a therapeutic antibody stream responsible for fouling virus filters consisted of monomeric variants containing more exposed hydrophobic surfaces.^16^ Hydrophobicity variants may self‐associate with other monomeric variants and lead to aggregates or could adsorptively foul the filtration membrane, leading to flux decay. Bieberbach et al.^17^ also reported two mechanisms of virus filter fouling: gross plugging by irreversible aggregates and decelerated mAb transport across the VF membrane caused by reversible aggregation (monomeric self‐association).^17^

This study used a mixed‐mode chromatography resin (Eshmuno CMX) to fractionate a bulk mAb pool into proteoform‐ and glycoform‐enriched sub‐pools for microvariant manufacturability assessment. Eshmuno CMX possesses carboxylate (COO—) moieties which impart weak cation exchange characteristics and alkyl functional groups which impart moderate hydrophobic interaction characteristics.^18^ This study applied a pH and shallow conductivity gradient to enhance the selectivity of Eshmuno CMX to the mAb proteoforms. The mixed‐mode combination provides a robust option for concurrent glycovariant, charge variant, and hydrophobicity variant fractionation. mAb 12 is a Chinese hamster ovary (CHO) cell‐derived IgG1 molecule. Before fractionation, mAb 12 harvest material was depth‐filtered, affinity‐captured using protein A resins, and polished using ion‐exchange chromatography steps. The fractionated pools yielded multiple manufacturability datasets corresponding to the relative amounts of different proteoforms, hydrophobicity variants, or glycoforms in each sub‐pool. This sub‐pool‐specific information is pertinent for difficult‐to‐filter mAbs and may help CMC teams identify fouling species, enabling selective depletion of these proteoforms. The aggregation propensity of each sub‐pool may provide an unconventional approach for early identification of proteoforms with poor manufacturability, enabling feedback to upstream production and drug development teams. Bioanalytical assays at this stage of sub‐pool manufacturability often include the diffusion interaction parameter (k_D_), relative hydrophobicity, high‐molecular‐weight (HMW) percent by size‐exclusion chromatography (SEC), glycan and charge variant profiling, and isoelectric point profiling.

## 2 MATERIALS AND METHODS

### 2.1 Materials

Tris base (MilliporeSigma cat# 648310), glacial acetic acid (MilliporeSigma cat# 695092), sodium hydroxide (MilliporeSigma cat# 567530), and sodium chloride (MilliporeSigma cat# S9888) were used for buffer preparation. Other materials used include 30‐kDa MWCO regenerated cellulose ultrafiltration discs (MilliporeSigma cat# PLTK07610), Amicon stirred cell 400 mL (MilliporeSigma cat# UFSC40001), and 0.22‐μm Stericup sterile single‐use bottle‐top filters (MilliporeSigma cat# S2GVU05RE). Eshmuno CMX mixed‐mode resin (MilliporeSigma cat# 1206500100) was packed in a Vantage L column (MilliporeSigma cat# 96160250). Viresolve Pro (VPro) Micro Devices (MilliporeSigma cat# VPMKVALNB9) were used for virus filtration. Bristol Myers Squibb (Devens, MA) provided a CHO cell‐expressed IgG1 molecule, identified as mAb 12 (reference pI = 8.2).

### 2.2 Methods

#### 2.2.1 Chromatography

Vantage columns packed with mixed‐mode resin (Eshmuno CMX) were used to fractionate ion exchange‐polished mAb 12. The mAb 12 feed was prepared in a low‐conductivity buffer (25 mM Tris base titrated with glacial acetic acid to pH 5, conductivity 1.4 mS/cm). A Vantage L column was packed with Eshmuno CMX resin to a volume of 37 mL (1.6 cm I.D. × 18.5 cm bed height). The equilibration buffer was 25 mM tris acetate (pH 6.7, 1.4 mS/cm), and the elution buffer was 50 mM tris acetate, 35 mM NaCl (pH 9.2, 4.2 mS/cm).

Chromatography recipes were prepared in Unicorn software for the ÄKTA Pure 25 to achieve mAb 12 fractionation. Step pH gradient elution was achieved by maintaining each pH step for two column volumes (CVs), starting with 10% elution buffer and increasing by 2% increments to 14%, followed by 3% increments to 35%. At that point, all peaks were collected. Further increments of 20% were applied up to 75%, followed by a 15% increment to 90% and a 10% increment to 100% in the elution buffer. Mixed‐mode resin (Eshmuno CMX) was selected due to the cationic and hydrophobic (mixed mode) membrane chemistries to improve the selectivity of mAb 12 proteoforms. The feed mAb was loaded at 4 g/L onto the column at a flow rate of 8 mL/min (linear velocity of 240 cm/h). The flow rate remained at 8 mL/min during the wash and elution steps.

Fractionation resulted in a plurality of proteoform‐enriched sub‐pools (A–I). Pre‐fractionation feed mAb 12 and post‐fractionation sub‐pools were subsequently buffer‐exchanged to a final buffer of 35 mM Tris acetate and 15 mM NaCl (pH 8 and conductivity of 2 mS/cm) at a concentration of 5 g/L before filterability evaluation on the VPro virus filter. An Amicon ultrafiltration stirred cell (400 mL) with 30 kDa MWCO regenerated cellulose ultrafiltration discs was used to concentrate the feed and sub‐pools, followed by buffer exchange using four diavolumes of reformulation buffer (35 mM Tris acetate, 15 mM NaCl, pH 8). Manufacturability assessment of fractionated sub‐pools was performed to show the most likely variants contributing to the rapid onset of VPro flux decay. VF flux decay profiles were shown in mass throughput (g/m^2^) units, a product of volumetric throughput and mAb concentration.

#### 2.2.2 Virus filtration and prefiltration

The feed fraction and fractionated sub‐pools A–I were filtered in a coupled sterile filtration mode using an Express SHC Optiscale‐25 0.22 μm sterile filter directly connected to a VPro virus filter. Viresolve Pro Micro 40 devices (VPro), with effective filtration areas of 3.4 cm^2^, were used to filter all fractions at 30 psi. An automated data acquisition (DAQ) system connected to a Mettler Toledo scale was used to acquire filtration data. Weight measurements were converted into mass throughput (g/m^2^), volumetric flux (L/m^2^.h), and normalized flux (J/J_0_).

#### 2.2.3 Size exclusion chromatography (SEC)

SEC was carried out using an Agilent 1100 HPLC (Agilent Technologies) equipped with a TOSOH TSKgel G3000SWXL column. The column was operated at a flow rate of 0.75 mL/min. The mobile phase comprised 0.05 M sodium phosphate and 0.3 M NaCl (pH 6.9).

#### 2.2.4 Dynamic light scattering to determine particle size, diffusion coefficient, and diffusion interaction parameter

The average hydrodynamic radius and diffusion interaction parameters (k_D_) for mAb 12 sub‐pools and feed fraction were determined by dynamic light scattering (DLS). Diffusion interaction parameter (k_D_) is a technique for studying weak protein–protein interactions.^19^ The hydrodynamic radius of mAb 12 fractions was measured at 1 g/L. Fractionated mAb sub‐pools were also serially diluted from 12 to 1 g/L in 35 mM tris acetate and 15 mM NaCl (pH 8) to measure the diffusion coefficients as a function of mAb concentration. Samples were centrifuged at 17000g for 10 min at 25°C, and 40‐μL aliquots were transferred to a 384‐well clear‐bottom microplate (Corning). Measurements at room temperature (25°C) were taken using a DynaPro PlateReader III (Wyatt), with ten 10‐s acquisitions scheduled and automated for each sample using Wyatt Technology Dynamics software. The data was averaged, and the diffusion interaction parameter was determined as follows:

$$\text{𝐷}=\text{𝐷𝑜}\;(1+{\text{κ}}_{\text{𝐷}}\text{𝑐)}$$

where 𝐷 is the diffusion coefficient, and 𝑐 is the concentration of the protein solution.^19^ Each sample was plated and measured in triplicate.

#### 2.2.5 Analytical hydrophobic interaction chromatography

The hydrophobicity of each fractionated mAb pool was evaluated by hydrophobic interaction chromatography (HIC) using a TSKgel Butyl‐NPR column (Tosoh Bioscience) installed on an Agilent 1200 series HPLC (Agilent Technologies) equipped with a fluorescence detector (excitation 340 nm, emission 280 nm) and UV–VIS diode array detector (DAD, 280 nm). The column was equilibrated with 2 M ammonium sulfate and 100 mM sodium phosphate (pH 7). Undiluted samples (50 μg) were directly injected onto the column and eluted over a 25‐min linear gradient of 100 mM sodium phosphate (pH 7) at a flow rate of 0.5 mL/min. The relative hydrophobicity was calculated from the areas under the curves of seven distinct sections of the chromatogram (V1‐V7).

#### 2.2.6 Imaged capillary isoelectric focusing

The charge heterogeneity of mAb 12 was determined by imaged capillary isoelectric focusing (icIEF) on a ProteinSimple Maurice instrument fitted with a ProteinSimple Maurice cIEF cartridge. Samples were prepared at a final concentration of 0.1 g/L in a mixture of 4% Pharmalyte pH 3–10 (GE Healthcare), 0.35% methylcellulose, 10 mM arginine, and 1% of the pI markers 4.05 and 9.90. Separation was achieved by applying pre‐focusing at 1.5 kV for 1 min and focusing at 3 kV for 6 min. UV absorbance was detected at 280 nm. Data were analyzed using Compass for iCE software (ProteinSimple). The charge heterogeneity of the samples was reported as a percentage of the total response, calculated from the area under the icIEF electropherogram curves.

#### 2.2.7 Glycan quantification

Enzymatic N‐glycan cleavage of mAb 12 (200 μg) was performed using the PNGase Fast Kit (Sigma‐Aldrich, St. Louis, MO). For every 10 μL of mAb 12, 2.5 μL of denaturing buffer and 0.5 μL of 1% 2‐Mercaptoethanol (BME) were added. The samples were denatured by incubating at 70°C for 10 min. Following denaturation, the mAb solution was filtered through a 30 kDa Amicon centrifuge filter (MilliporeSigma, Burlington, MA) and washed with 50 mM ammonium bicarbonate by centrifugation at 14,000 rpm for 5 min. Subsequently, 1 μL of PNGase F enzyme solution was added, and the samples were incubated at 37°C for 1 h. After incubation, the samples were centrifuged again using the 30 kDa Amicon filter to capture the released glycans. The released glycans were concentrated by vacuum evaporation for 1 h and 15 min to prepare them for HPLC analysis. The N‐glycans were processed using Agilent's GlykoPrep 2‐AB Labeling and Cleanup Modules (Agilent Technologies, Santa Clara, CA).

N‐glycan separation was performed using an Agilent 1290 UHPLC system equipped with an ACQUITY UPLC Glycan BEH Amide column (Waters, Milford, MA) maintained at 60°C. The column was initially equilibrated with 100% acetonitrile, followed by a linear gradient of 100 mM ammonium formate at pH 4, from 24% to 40% over 32 min to elute the N‐glycans. Detection was performed with a fluorescence detector set to 330 nm excitation and 420 nm emission wavelengths. The percentage of N‐glycans was calculated from the areas under the respective peaks in the chromatogram. N‐glycan peak identification was achieved by running standards for G0F‐N, G0, G0F, G1a, G1b, G1Fa, G1Fb, and G2F (Agilent) to establish a glycan library for comparison.

#### 2.2.8 Circular dichroism (CD) spectroscopy

An Applied Photophysics ChiraScanV100 CD Spectrometer was used to perform far‐UV CD spectroscopy (185–260 nm). All measurements were taken at 20 degrees Celsius, and the cell path length was 0.05 cm. The concentration of mAb 12 feed and sub‐pool fractions was 5.0 mg/mL. The formulation buffer was 35 mM Tris acetate and 15 mM NaCl at pH 8, with a conductivity of 2 mS/cm. ProtaCal software (CD Module) was used for data analysis. All spectra were processed using buffer subtraction and normalization, followed by calculation of secondary structure composition in each sub‐pool and feed mAb 12 fractions. Far‐UV CD probes the peptide backbone chain, revealing secondary structural composition of proteins such as alpha‐helices, beta‐sheets, bends, turns, and others.^20^, ^21^

#### 2.2.9 Fourier‐transform infrared (FT‐IR) spectroscopy

Spectra were collected on an FT‐IR spectrometer (PROTA‐3S with MCT detector) over 4000–800 cm^−1^ at 4 cm^−1^ spectral resolution. The collection time was 200 scans in 4 min. Aliquots were measured at 25°C using attenuated total reflection (ATR) with a single bounce diamond crystal. Background spectra (air/clean crystal or empty cell) were acquired immediately before each sample. The instrument compartment was purged with dry nitrogen throughout. Data were exported as absorbance (a.u.). Each sample was equilibrated for ~1 min before acquisition. Spectra were trimmed to 2000–800 cm^−1^ and baseline‐corrected using a second‐order Savitzky Golay (5‐point) polynomial fit to the full region. In the Savitzky–Golay smoothing technique, a sliding window of five data points was used to sequentially fit the second order polynomial, effectively reducing noise while preserving signal peaks.

## 3 RESULTS AND DISCUSSION

The platform‐based purification and polishing of mAbs are suitably templated with cation‐exchange bind‐and‐elute and anion‐exchange flow‐through polishing steps. A pH step gradient may be used instead of the more common linear salt gradient during bind‐and‐elute cation exchange or mixed‐mode chromatography.^22^ A pH step gradient causes the elution of bound mAb variants in order of surface charge or pI, surface hydrophobicity, or size (fragment, monomer, or oligomer).^23^ Small‐step pH gradients (0.1 pH units) with mild increases in conductivity have previously been reported to resolve multiple IgG1 proteoforms distinctly.^24^

### 3.1 Chromatographic fractionation

**FIGURE 1:**
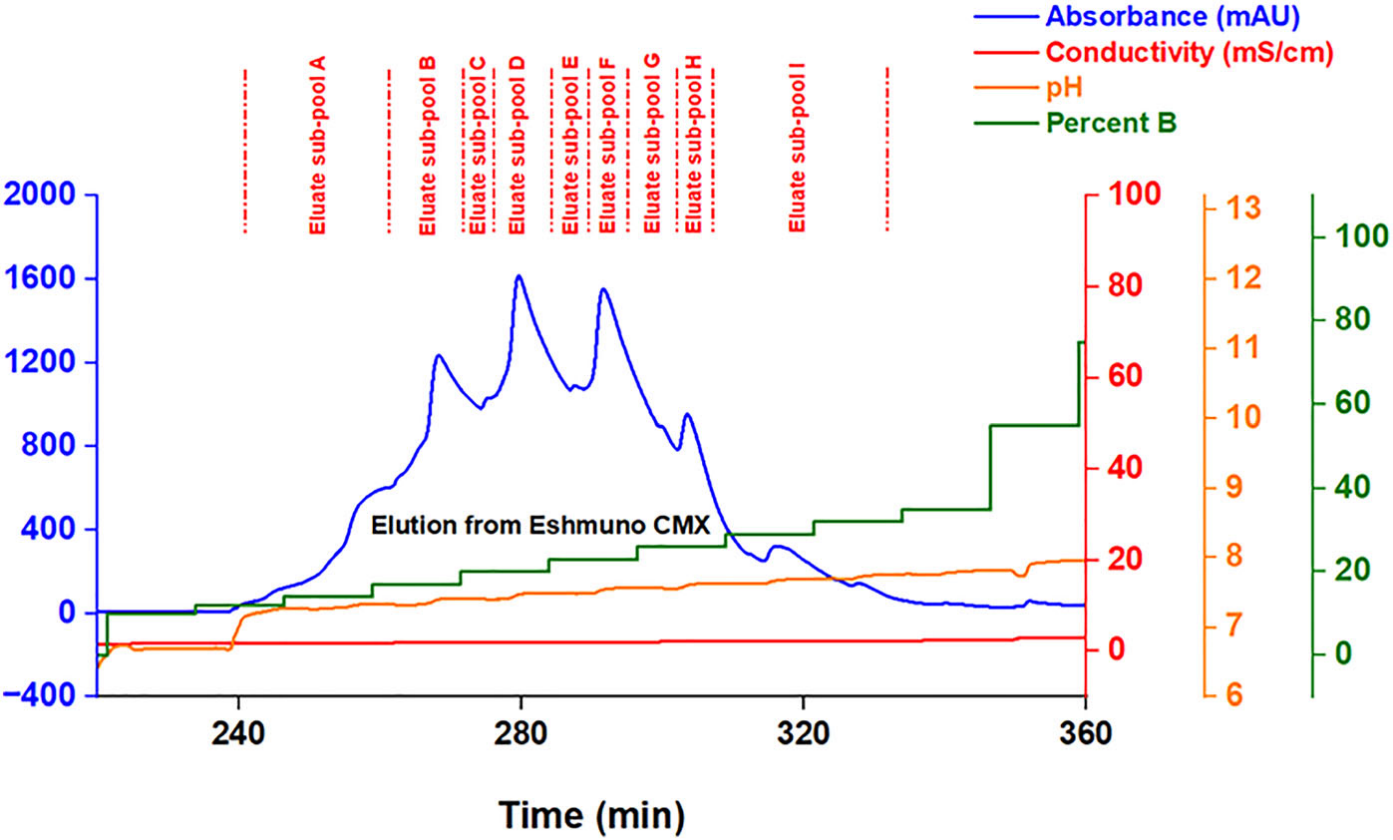
Chromatographic traces showing fractionation cutoffs and the elution strategy for the step pH gradient fractionation of mAb 12 from a Vantage column packed with 37 mL of Eshmuno CMX resin.

In this study, step elution using pH gradients and mild conductivity differences between equilibration buffer A (25 mM tris acetate; pH 6.7, 1.4 mS/cm) and elution buffer B (50 mM tris acetate; pH 9, 4.2 mS/cm) was used to enrich different mAb 12 proteoforms and variants on Eshmuno CMX resin. This mAb was formerly purified using platform purification and polishing steps to eliminate host cell proteins and non‐mAb impurities. A Vantage column was packed with 37 mL of Eshmuno CMX (mixed‐mode weak cationic plus moderate HIC resin). Figure 1 shows the zoomed‐in elution section of the chromatogram during the step elution of mAb 12 from the 37 mL Eshmuno CMX column. The entire chromatogram is shown in Figure S1.

Forward pH‐gradient step elution using Eshmuno CMX resin yielded multiple peaks corresponding to different enrichment levels of mAb 12 variants. The mixed‐mode mechanism enhanced the selectivity of Eshmuno CMX resin for these proteoforms. Hydrophobic ligands contribute to the enrichment of more hydrophobic variants as elution time increases.^25^ Weak cationic ligands enable the early elution of acidic charge variants and the late elution of basic charge variants.^26^ Each eluate sub‐pool was buffer‐exchanged and concentrated using Amicon stirred cells fitted with 30 kDa MWCO ultrafiltration discs, with four diavolumes before testing for filterability on the VPro virus filter. Nine eluate sub‐pools (A–I) were collected separately for manufacturability assessment.

### 3.2 Size exclusion chromatography and dynamic light scattering

**FIGURE 2:**
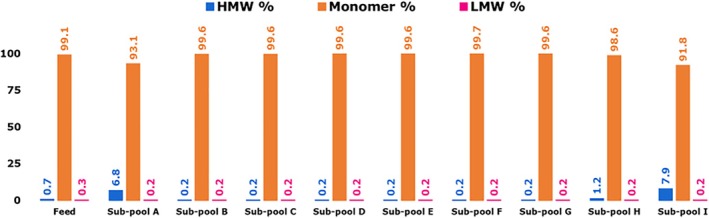
Size exclusion chromatography (TSKgel G3000SWXL) for the quantification of high‐molecular‐weight (HMW) aggregates, monomeric mAb, and low‐molecular‐weight (LMW) mAb fragments for feed mAb 12 and sub‐pools eluted from Eshmuno CMX resin‐packed column.

Sub‐pools A‐I and bulk mAb 12 feed were assayed for higher molecular weight species and lower molecular weight species using size exclusion chromatography. Figure 2 shows the SEC molecular composition for mAb 12 feed and sub‐pools eluted from Eshmuno CMX resin.

The proportion of high‐molecular‐weight (HMW) species was constant at 0.2% from sub‐pools B to G before increasing to 1.2% and 7.9%, respectively at sub‐pools H and I. An increase in HMW for sub‐pools H and I was offset by the decrease in monomeric mAb. Sub‐pool A showed a HMW percent of 6.8%. Unfractionated bulk mAb 12 feed contained 0.7% HMW species, sub‐pool H showed a 1.71x increase in HMW, and sub‐pool I showed an 11x increase in HMW compared to the feed fraction.

The proportion of low‐molecular‐weight (LMW) species never exceeded 0.3% across all fractions, indicating that LMW species may not have significantly contributed to fouling of the VPro filters. The proportion of monomeric mAb decreased from 99.1% in the feed to 91.8% in sub‐pool I. The contribution of HMW aggregates to virus filter fouling is expected to be highest in sub‐pool I, followed closely by sub‐pool A. Other factors may contribute to VPro filter fouling beyond gross plugging by aggregates. Size‐based prefilters are designed to remove large aggregates with sizes exceeding 0.1 μm.^27^

**FIGURE 3:**
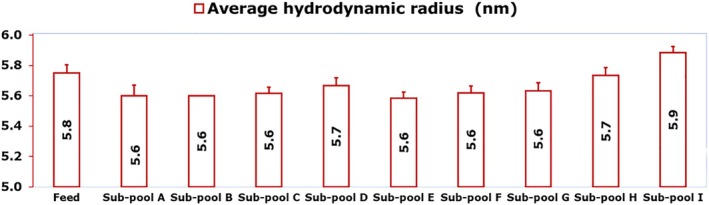
Dynamic light scattering for determination of the average hydrodynamic radius of mAb variants in the feed fraction and eluate sub‐pools from Eshmuno CMX resin‐packed column.

Dynamic light scattering was used to determine the average hydrodynamic radius of mAb 12 feed and fractionated pools in identical buffer conditions (35 mM tris acetate, 15 mM NaCl, pH 8), and the result is shown in Figure 3.

The average hydrodynamic radius of mAb 12 molecules increased slightly from 5.6 nm in sub‐pool A, and most other sub‐pools, to 5.9 nm in sub‐pool I. The average hydrodynamic radius in sub‐pool I exceeds that of the feed mAb by 0.1 nm. An increase in the average hydrodynamic radii of proteins in solution is indicative of greater aggregation and molecular self‐association.^28^, ^29^ Sub‐pool I was the most fouling fraction on the VPro virus filter, and its high average hydrodynamic diameter partly explains this.

**FIGURE 4:**
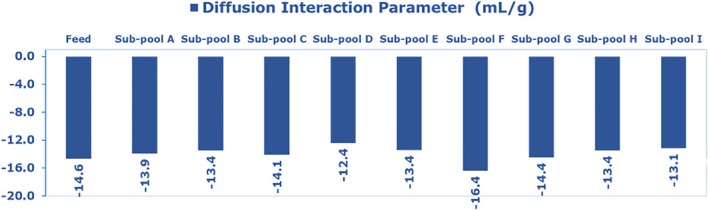
Diffusion interaction parameter (k_D_) values for the feed fraction and sub‐pools of mAb 12 eluted from the Eshmuno CMX resin‐packed column.

Dynamic light scattering was also used to determine the diffusion interaction parameter (k_D_) of mAb 12 sub‐pools. The diffusion interaction parameter k_D_ is indicative of colloidal stability and intermolecular self‐association.^30^, ^31^, ^32^ A negative k_D_ indicates the prevalence of intermolecular attractive forces (monomeric self‐association), a precursor of reversible aggregation, while a positive k_D_ indicates intermolecular repulsion forces.^19^, ^33^ Figure 4 shows the k_D_ values of mAb 12 feed and eluate sub‐pools A–I.

The k_D_ value was highly negative across sub‐pools A–I. The k_D_ value for sub‐pools A–I stayed in the range of −13 to −16.4 mL/g. The feed fraction also showed a highly negative k_D_ value of (−14.6 mL/g). The implication of slight differences in k_D_ (+/− 2 mL/g) among sub‐pools A–I may be that different mAb 12 variants possess similar potential for aggregation via the monomeric self‐association mechanism (reversible aggregation). Collectively considering all the bioanalytical assays in this work, k_D_ is possibly one factor out of several that account for differences in filterability between sub‐pools. However, the highly negative k_D_ value sufficiently explains the general filterability challenge across all mAb 12 sub‐pools, as well‐behaved antibody formulations typically show k_D_ values greater than −3 mL/g, such as a value of −1.2 mL/g.^24^ A protein with a positive k_D_ value will have good colloidal stability and intermolecular net repulsion.^19^, ^34^

### 3.3 Relative hydrophobicity, CD spectroscopy, and FT‐IR spectroscopy

**FIGURE 5:**
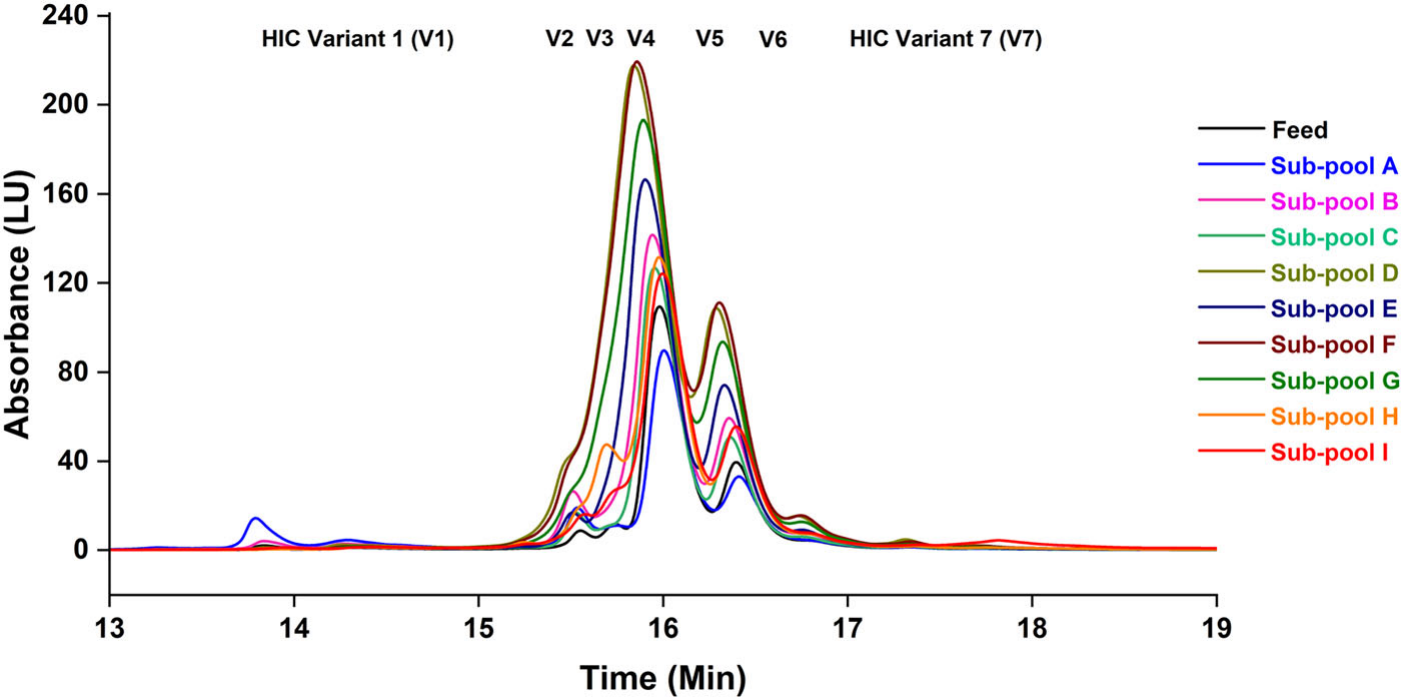
TSKgel Butyl‐NPR analytical hydrophobicity variant profile for sub‐pools of mAb 12 eluted from Eshmuno CMX resin‐packed column.

**FIGURE 6:**
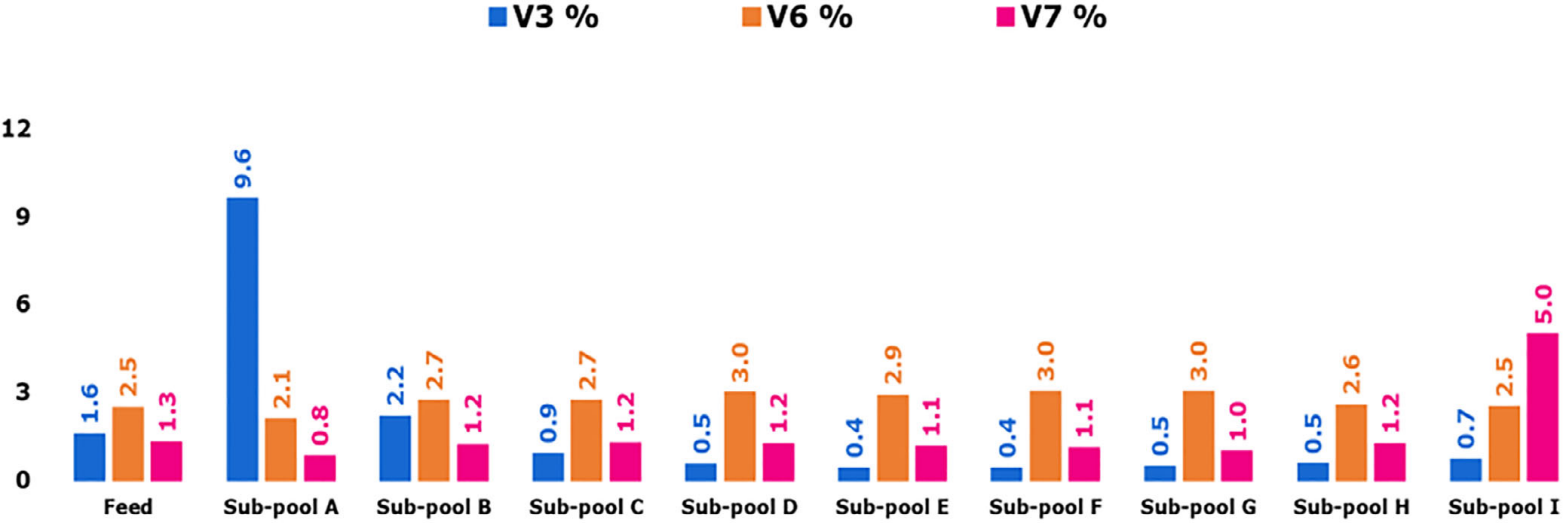
TSKgel Butyl‐NPR analytical hydrophobicity variant profiles (variant 3 or V3, variant 6 or V6, and variant 7 or V7) for sub‐pools of mAb 12 from Eshmuno CMX resin‐packed column.

The feed fraction and eluate sub‐pools of mAb 12 from the Eshmuno CMX column were characterized using an analytical HIC column, and the retention time was used as a metric of relative hydrophobicity. Figure 5 shows the hydrophobicity variant profiles and peak designations (V1 for the least hydrophobic mAb 12 variants to V7 for the most hydrophobic mAb 12 variants) of the feed and eluate sub‐pool fractions. Table S1 summarizes the hydrophobicity variant peak integrations to quantify these HIC variants per sub‐pool. The percentages of notably varying hydrophobicity variants V3, V6, and V7 in the feed and eluate sub‐pools are shown in Figure 6.

Hydrophobicity variants of mAb 12 were separated using the TSKgel butyl NPR analytical column. A trend of increasing hydrophobicity was observed from sub‐pool A to sub‐pool I. The least hydrophobic variants (V1, V2, and V3) were enriched in sub‐pool A. In contrast, the most hydrophobic variant (V7) was enriched in sub‐pool I. The retention time of V7 was about 4 min longer than that of V1, indicating that V7 is much more hydrophobic than V1. Sub‐pool I showed a V7 percentage of 5% (3.85× enrichment over the feed fraction). In contrast, sub‐pool A showed a V7 ratio of 0.8% (0.62× depletion over the feed fraction). Sub‐pool I also showed a lower percentage of the less hydrophobic variant V3 (0.7%) compared with sub‐pool A (9.6%). The enrichment of the most hydrophobic variant (V7) in sub‐pool I may partly account for the filterability outcome (final throughput) on the VPro virus filter. Other product quality parameters, including HMW species, also play a part in filterability. Differences in mAb higher‐order structure determine the relative hydrophobicity of mAb molecules.^35^

**FIGURE 7:**
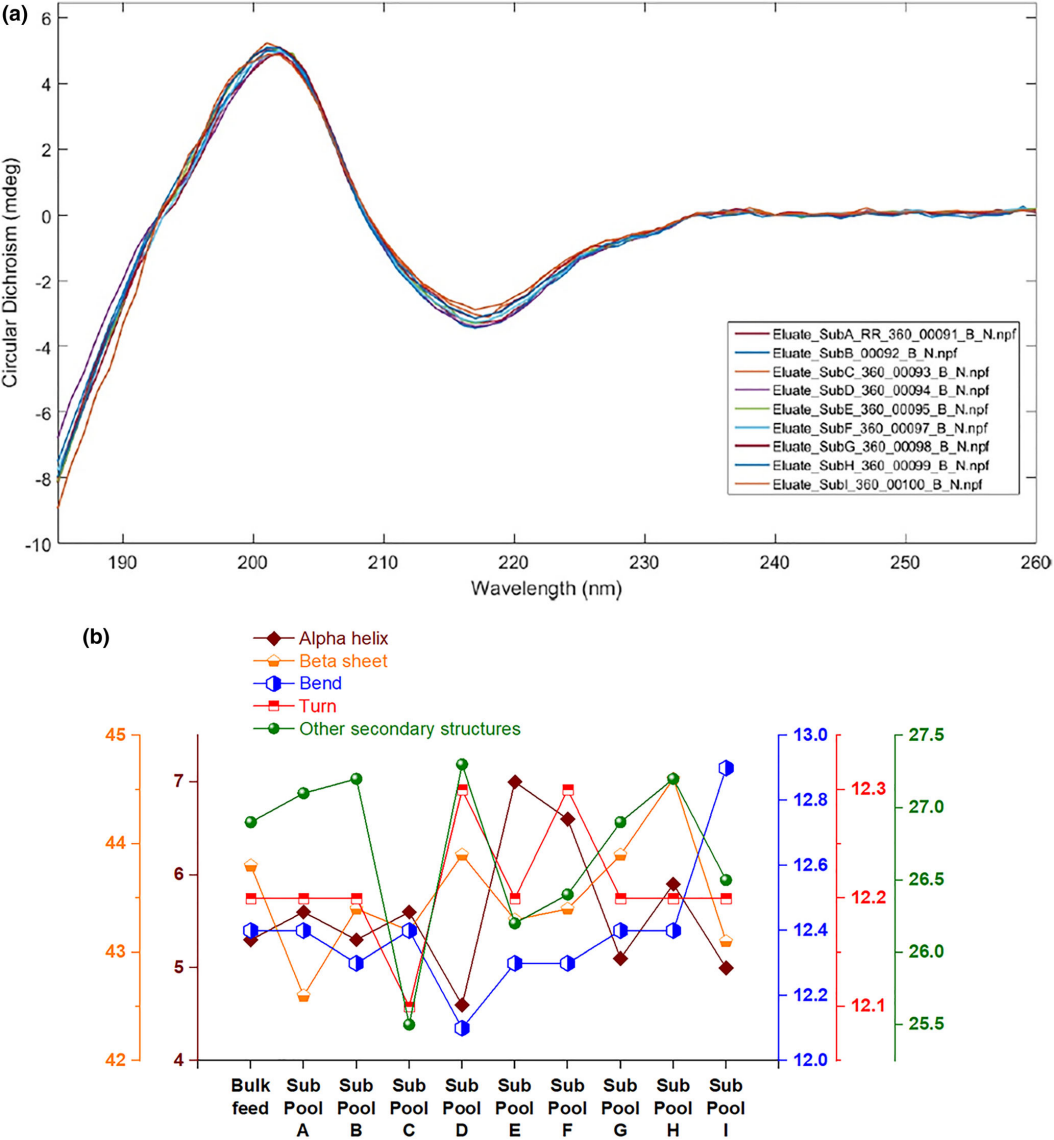
(a) CD spectra for sub‐pools of mAb 12 eluted from Eshmuno CMX resin‐packed column. (b) Secondary structure results for mAb 12 feed and eluate sub‐pools from Eshmuno CMX resin‐packed column.

CD spectroscopy was used to quantify the secondary structural composition of mAb 12 feed fraction and sub‐pools. The goal of this assay was to highlight the ratios of alpha helices, beta sheets, and other secondary structures in the fractionated pools. Figure 7a shows the CD spectra of mAb 12 sub‐pools, while Figure 7b shows the calculated values of all secondary structure components using the ProtaCal software.

CD spectra show that there was significant similarity in secondary structure across all mAb 12 fractions, confirming that any heterogeneity between the mAb variants is not at the macro‐scale but at the micro‐scale. Results from the ProtaCal software show some increase in the percentage of alpha helices from sub‐pools D–F, with values of 4.6%, 7.0%, and 6.6% respectively, before dropping to a value of 5.0% in sub‐pool I. Sub‐pool I also showed the highest percentage of bends (12.9%) when compared to the other sub‐pools and the feed fraction (averaging 12.4% bends). Secondary structures (especially bends or turns, helices, strands, and sheets) play a significant role in determining the final functional conformation of the antibody variant.^20^, ^21^ Small differences in secondary structure composition may translate to more noticeable differences at the quaternary structure level, such as the exposure of hydrophobic patches.

**FIGURE 8:**
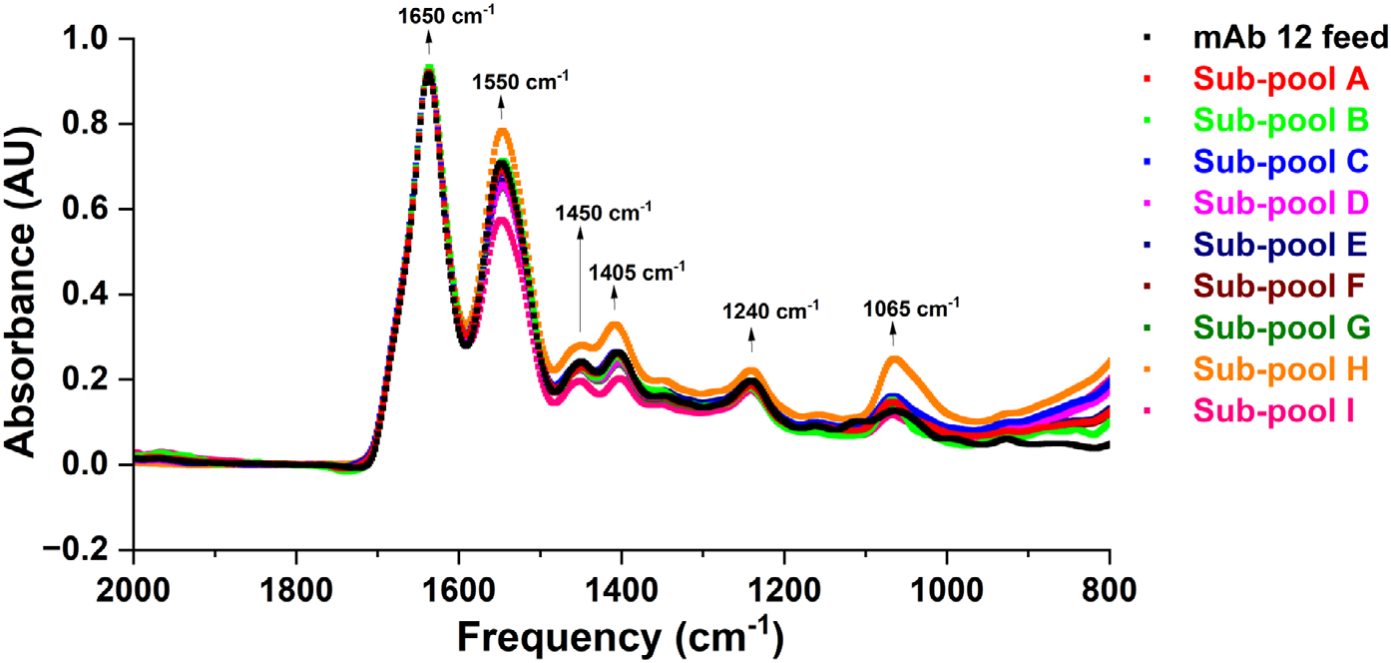
FT‐IR spectra for feed fraction and sub‐pools of mAb 12 from Eshmuno CMX resin‐packed column.

FT‐IR spectroscopy was performed as an orthogonal method for secondary structure determination of mAb 12 feed and eluate sub‐pools. The results are shown in Figure 8.

FT‐IR spectra show that there was no significant downshift in the amide I and amide II bands (1650 and 1550 cm^−1^, respectively) for the feed and eluate fractions. The amide I band of all eluate and feed fractions perfectly overlaps and this peak is because of C=O stretching vibrations of the peptide bond.^36^ The amide II band showed slightly different absorbance values, but the wavenumber (frequency) remained the same, an indication of similarity in secondary structure. In totality, FT‐IR was not able to sufficiently show structural differences between the mAb variants enriched in each sub‐pool; thereby, reinforcing the claim that molecular heterogeneities among variants were of a microheterogenous nature.

### 3.4 Glycan and charge variant analyses

**FIGURE 9:**
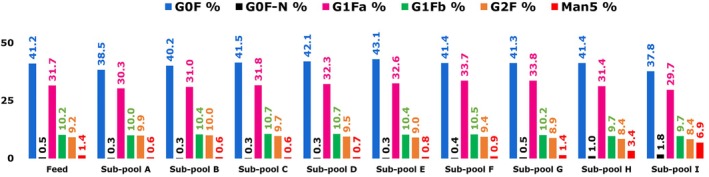
Glycan profile for feed and sub‐pools of mAb 12 eluted from Eshmuno CMX resin‐packed column.

Glycan assays and charge variant analyses were performed for the unfractionated feed mAb 12 and the fractionated sub‐pools. Figure 9 shows the percentage of major glycans in sub‐pools of mAb 12 eluted from the Eshmuno CMX resin. The complete list of all glycans in all mAb 12 fractions is shown in Table S2.

The mannose‐5 (Man‐5) glycovariant proportion increased progressively from 0.6% in sub‐pool A to 6.9% in sub‐pool I. Man‐5 glycoform accounted for 1.4% of the mAb 12 feed fraction. G0F‐N was also progressively enriched with increasing elution time from 0.3% in sub‐pool A to 1.8% in sub‐pool I. Among all the glycovariants in Figure 9 and Table S2, only G0F‐N and Man‐5 showed clear and consistent enrichment during the bind‐and‐elute fractionation of mAb 12. Several authors have outlined how appended glycan type may affect a protein's surface conformation, thereby promoting small changes in surface charge and hydrophobicity.^37^, ^38^, ^39^

**FIGURE 10:**
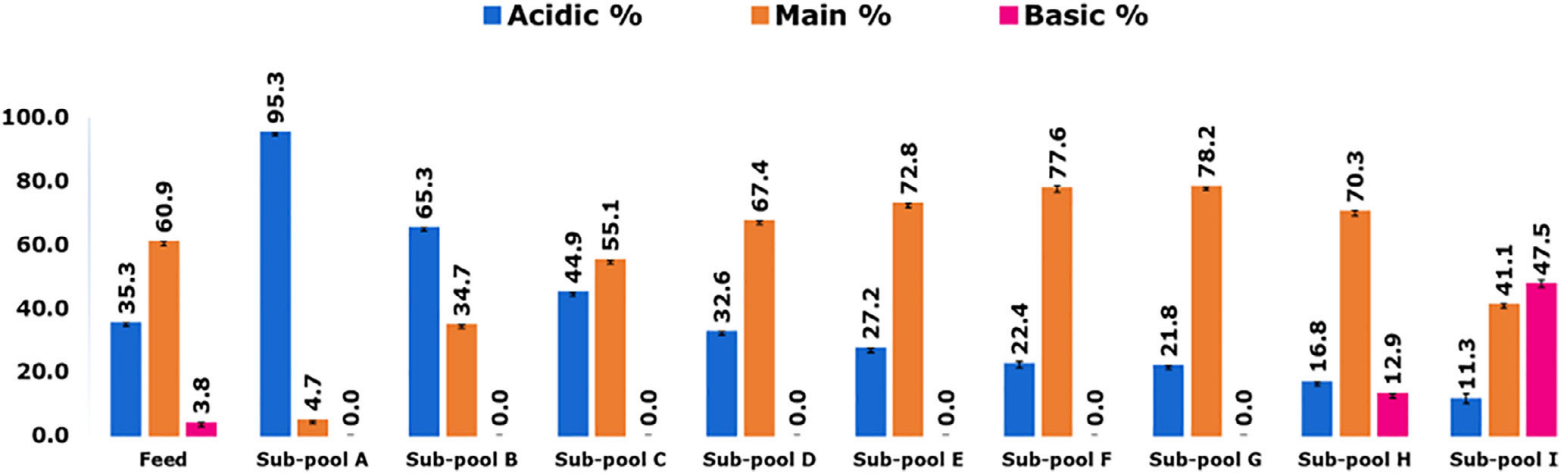
Charge variant profile for sub‐pools of mAb 12 sequentially eluted from Eshmuno CMX resin‐packed column.

The charge variant profile of mAb 12 feed and eluate sub‐pools was determined by icIEF, and shows the distribution of acidic, main (neutral), and basic charge variants in the feed and eluate sub‐pools of mAb 12. The results are shown in Figure 10.

The proportion of acidic charge variants decreased with elution order, whereas the proportion of neutral and basic charge variants increased. Eluate sub‐pool A consisted of 95.3% acidic charge variants, but this dropped to 11.3% in sub‐pool I, which contained 47.5% basic charge variants. The mixed‐mode chemistry of Eshmuno CMX resin enables the cationic resolution of charge variants, which is complemented by the additional hydrophobic selectivity of variants conferred by the hydrophobic functional groups on the resin.

### 3.5 Virus filtration

**FIGURE 11:**
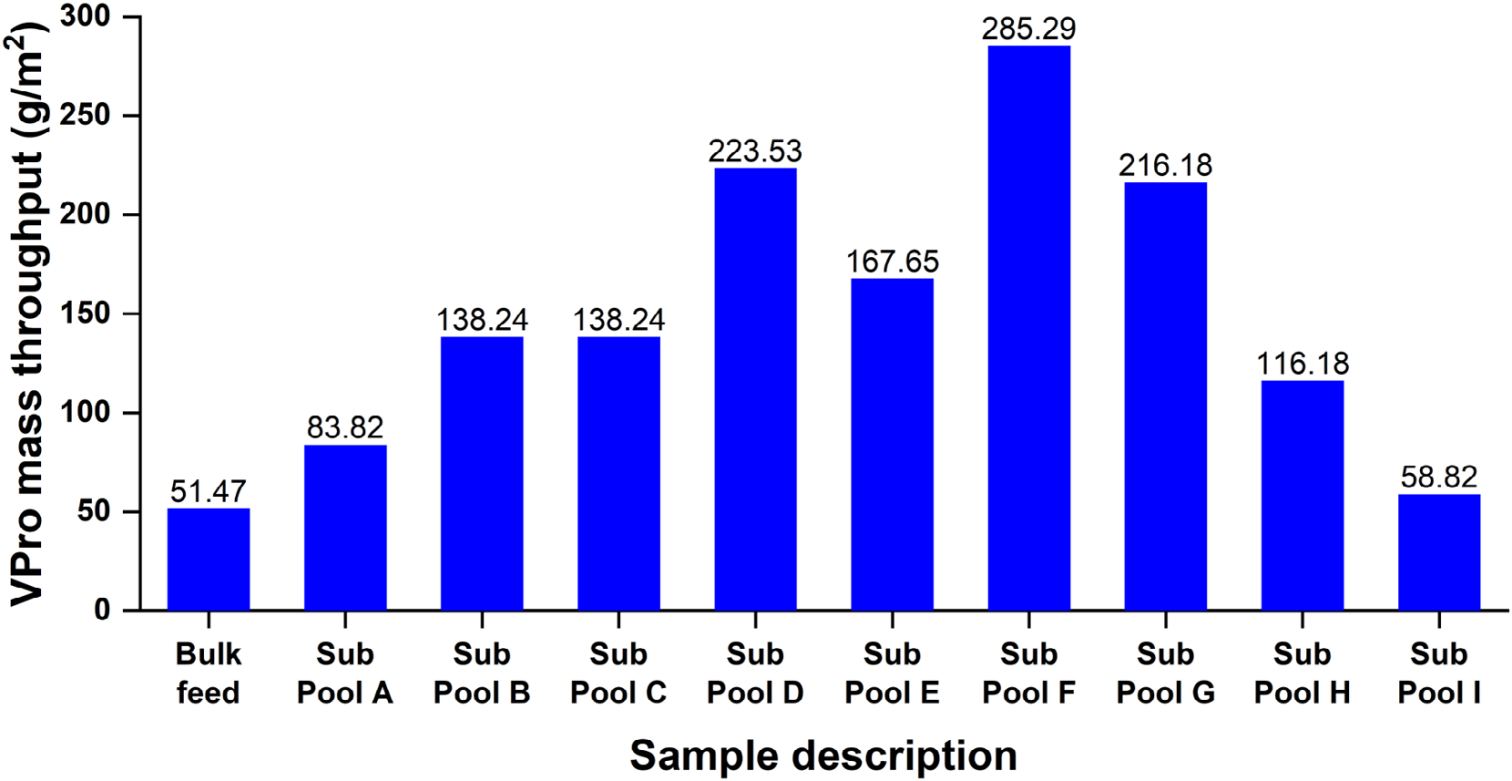
Final mass throughput of mAb 12 unfractionated feed (5 g/L in 35 mM tris acetate, 15 mM NaCl; pH 8) and mAb 12 sub‐pools A–I (5 g/L in 35 mM tris acetate, 15 mM NaCl; pH 8) filtered through the VPro virus filter without adsorptive prefiltration.

Virus filtration of the sub‐pools was performed without adsorptive prefiltration. A standardized filterability study of the bulk unfractionated mAb 12 feed and the fractionated sub‐pools A‐I was performed by coupling a 25 mm diameter Express SHC (Optiscale‐25) 0.22 μm sterile filter directly to VPro virus filters. The final mass throughput (obtained after a standard filtration time of 60 min and corresponding to over 95% flux decay) for each sub‐pool and feed fraction through the VPro filter was determined and replotted in Figure 11.

Rapid‐onset VPro membrane fouling with the unfractionated mAb 12 bulk feed was observed with 51.47 g/m^2^ of the feed filtered under bulk mAb conditions. Bulk mAb 12 was designated a difficult‐to‐filter molecule. Following the bulk fractionation, variable filterability was observed across the proteoform‐enriched sub‐pools. The mAb 12 mass throughput was higher in the centrally located sub‐pools (B–G), with a slight decline in throughput at sub‐pool H. Sub‐pool A and sub‐pool I showed significant fouling on the VPro virus filter. Sub‐pools D, E, F, and G show a threefold to sixfold enhancement in filterability through the VPro filter relative to the bulk feed mAb. All sub‐pools were formulated in 35 mM tris acetate, 15 mM NaCl (pH 8), a buffer condition designed to be as close as possible to the nominal pI of this molecule and promote moderate fouling during filtration.^40^

**FIGURE 12:**
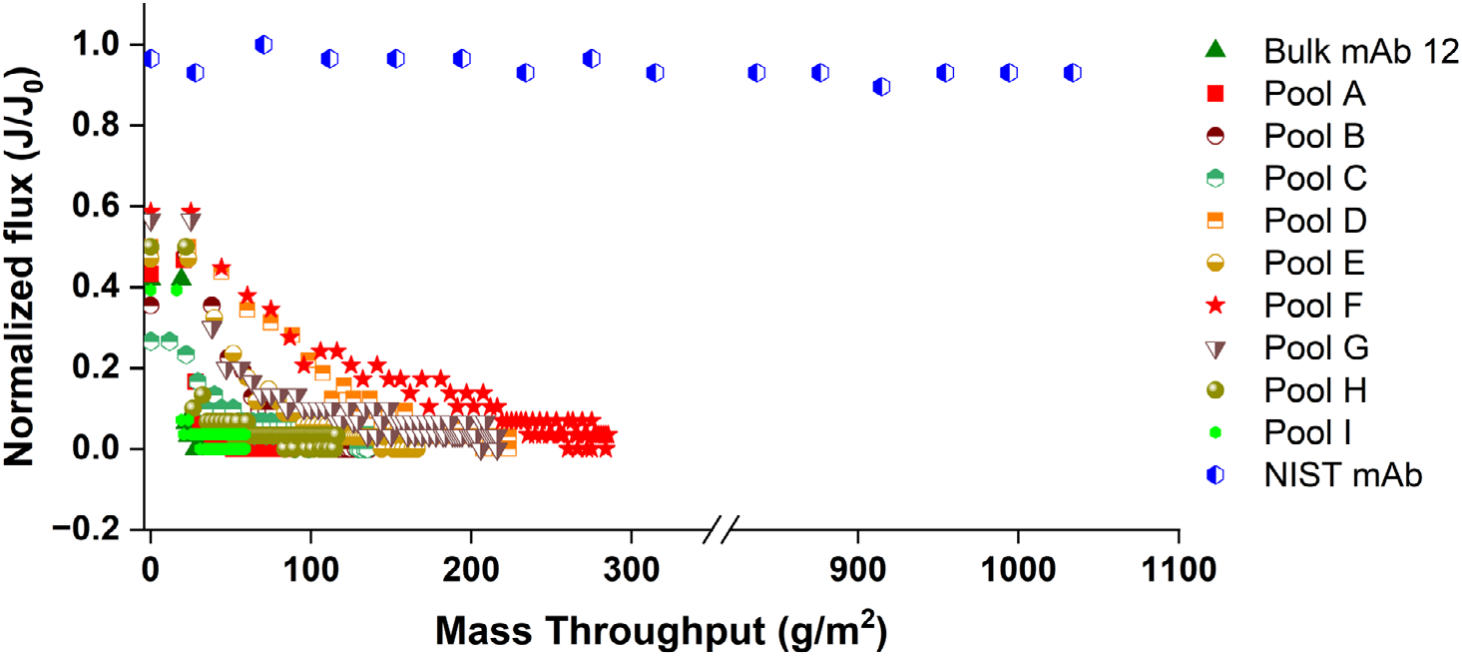
Normalized flux profiles plotted against mass throughput of bulk unfractionated mAb 12, fractionated sub‐pools of mAb 12 (5 g/L in 35 mM tris acetate, 15 mM NaCl; pH 8) and bulk unfractionated NIST mAb across an Express SHC Optiscale‐25 0.22 μm sterile filter directly coupled to a Viresolve Pro virus filter.

Sub‐pool filterability was evaluated in tandem with bioanalytical measurements to identify problematic product‐related variants that are either enriched or depleted across the various fractionated sub‐pools. Sub‐pools showing the most VPro fouling may be inferred to contain a higher percentage of aggregation‐prone mAb variants. Figure 12 presents the complete VPro filtration data for the bulk unfractionated mAb 12 and fractionated sub‐pools without adsorptive prefiltration. A reference plot containing filtration data of NIST mAb formulated at 5 g/L in the same buffer condition as mAb 12 is also included in Figure 12 for comparison.

Rapid flux decay of mAb 12 feed and eluate sub‐pools is shown alongside a well‐behaved NIST mAb sample. mAb 12 is a difficult‐to‐filter molecule, and the enrichment of mAb 12 variants in different sub‐pools provides incremental throughput improvement, although not of the magnitude shown by the filtration behavior of a well‐behaved antibody molecule. Collectively evaluating the product quality and VPro filterability results, a molecule may require clone re‐selection or bioreactor optimization when there is a significant deviation from desirable quality attributes. Downstream process development may sufficiently address low deviations from desirable product quality attributes. Chromatography polishing operations are typically used to remove HMW species, denatured species, or certain charge variants.

## 4 CONCLUSION

Rapid bioanalytical assessment of a drug candidate's quality attributes may help predict a difficult‐to‐filter molecule prior to confirming flux decay during virus filtration. Downstream teams may then provide feedback to upstream teams regarding desirable biophysical and bioanalytical attributes. Some product quality attributes may be addressed during the chromatography polishing and purification steps using peak cutting or prefiltration to eliminate problematic mAb variants. Downstream purification teams can deplete these aggregation‐prone species, especially low‐abundance problematic proteoforms such as Man‐5 glycoforms or outlying HIC variants such as V7. Biomanufacturing companies may use information from a fractionation study to identify problematic proteoforms for virus filters, especially the most hydrophobic proteoforms, certain glycovariants, or specific aggregation‐susceptible monomeric variants.

A new approach to understand the manufacturability bottleneck of mAb 12 through proteoform enrichment in sub‐pools was evaluated, and the manufacturability of individual sub‐pools was assessed using a combination of bioanalytical and filtration methods. The k_D_ of mAb 12 sub‐pools tended to be highly negative (below −13 mL/g) across the entire elution range, inferring a high propensity for intermolecular self‐association. The dominant fouling mechanism of mAb 12 is suggested to be the consistently high relative hydrophobicity of all variants (inferred by the retention time on a HIC column and benchmarked to in‐house standard molecules). Enrichment of the most hydrophobic variant of mAb 12 (V7) strongly correlated with the fouling propensity of sub‐pool I. The virus filter fouling mechanism of mAb 12 is concluded to be a combination of specific hydrophobically driven aggregation mechanisms, gross plugging by HMW species (SEC), and reversible aggregation (highly negative k_D_ across all sub‐pools). Future research should seek to show the impact of depleting certain variants on biological function (inhibition or enhancement of drug activity).

## AUTHOR CONTRIBUTIONS

**Solomon Isu:** Conceptualization; investigation; writing—original draft; methodology; visualization; writing—review and editing; formal analysis; data curation. **Derek Silva:** Investigation; writing—original draft; methodology; validation; visualization; data curation. **Melissa Holstein:** Conceptualization; funding acquisition; writing—review and editing; project administration; resources; supervision. **Angela Lewandowski:** Supervision; resources; project administration; writing—review and editing; funding acquisition. **Kristina Cunningham:** Resources; supervision; data curation; formal analysis; visualization; writing—review and editing. **Adam Sokolnicki:** Writing—review and editing; supervision; formal analysis; project administration; funding acquisition; conceptualization; resources. **Bala Raghunath:** Resources; supervision; formal analysis; writing—review and editing; funding acquisition; project administration.

## FUNDING INFORMATION

This research was performed using resources owned by either MilliporeSigma, Burlington, MA or by Bristol Myers Squibb, Devens, MA. No specific funding from either company or any other organization was provided for this research.

## CONFLICT OF INTEREST STATEMENT

The authors declare no conflicts of interest.

## Supporting information


**FIGURE S1.** Chromatographic traces showing the bind‐and‐elute chromatography of mAb 12 from a Vantage column packed with 37 mL of Eshmuno CMX resin.
**Table S1.** Integrated peaks for hydrophobicity variants V1–V7 in each mAb 12 sub‐pool.
**Table S2.** Glycan profile for feed fraction and sub‐pools of mAb 12 eluted from Eshmuno CMX mixed‐mode resin.

## Data Availability

The data that support the findings of this study are available from the corresponding author upon reasonable request.
