# Supplementary material for: A case study showing the role of hydrophobicity variants and other enriched mAb proteoforms on filterability through a virus filter with productivity improvement measures
Source: Biotechnol Prog. 2026 Jan 19;42(2):e70101. doi: 10.1002/btpr.70101 (PMC13055118; doi:10.1002/btpr.70101)
Supplement: Supplementary file 1 — FIGURE S1. Chromatographic traces showing the bind‐and‐elute chromatography of mAb 12 from a Vantage column packed with 37 mL of Eshmuno CMX resin. Table S1. Integrated peaks for hydrophobicity variants V1–V7 in each mAb 12 sub‐pool. Table S2. Glycan profile for feed fraction and sub‐pools of mAb 12 eluted from Eshmuno CMX mixed‐mode resin. [file BTPR-42-e70101-s001.docx]

**SUPPLEMENTARY FIGURES**

**
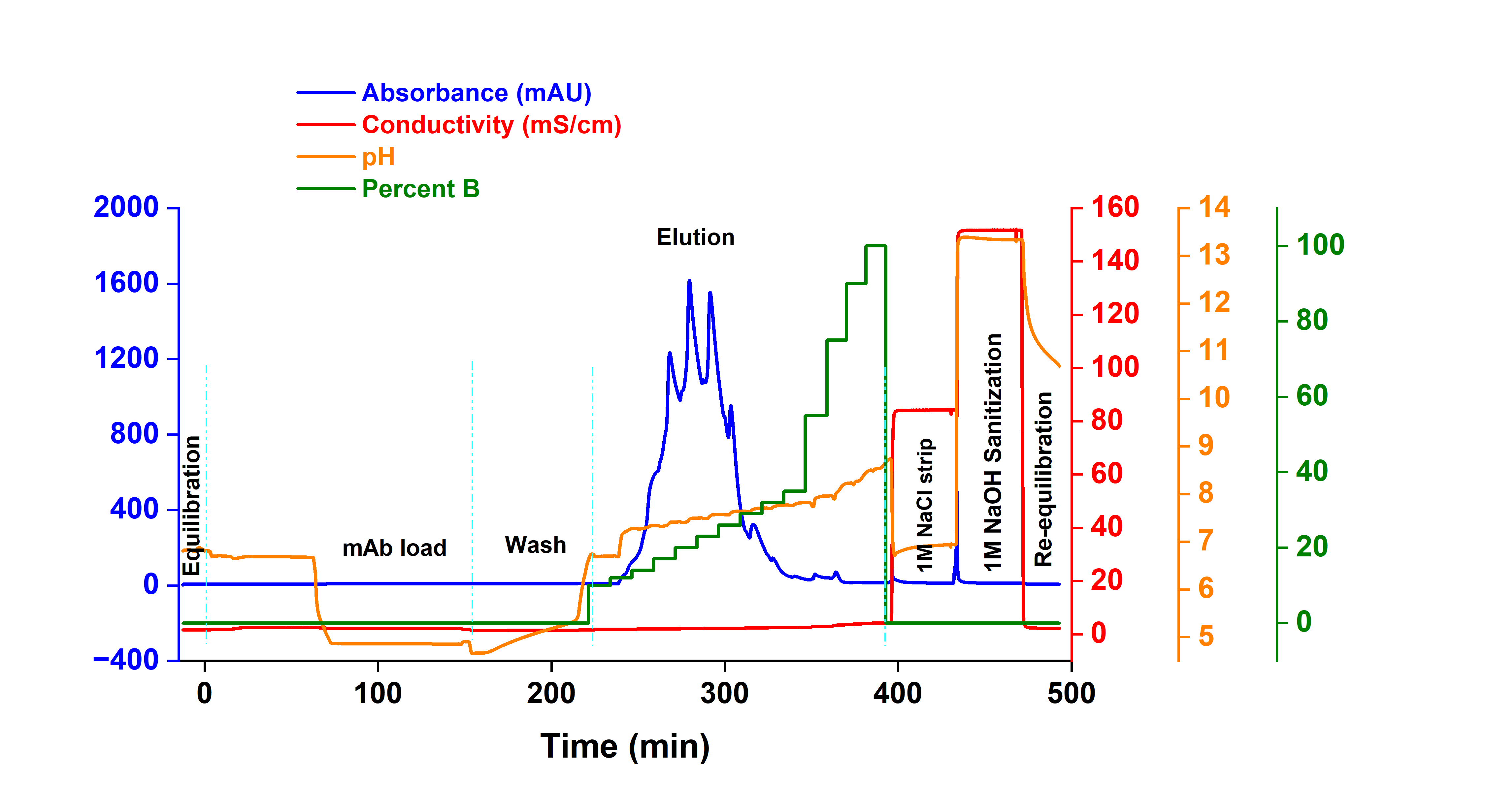
**

**FIGURE S1** Chromatographic traces showing the bind and elute chromatography of mAb 12 from a Vantage column packed with 37 mL of Eshmuno CMX resin.

**SUPPLEMENTARY TABLES**

**TABLE S1** Integrated peaks for hydrophobicity variants V1–V7 in each mAb 12 sub-pool.

| Sample Description | **HIC variant V1** | **HIC variant V2** | **HIC variant V3** | **HIC variant V4** | **HIC variant V5** | **HIC variant V6** | **HIC variant V7** |
| --- | --- | --- | --- | --- | --- | --- | --- |
| Feed mAb 12 | 0.44 | 0.06 | 1.55 | 71.51 | 22.70 | 2.46 | 1.27 |
| Sub-pool A | 0.64 | 0.32 | 9.60 | 65.97 | 20.61 | 2.06 | 0.81 |
| Sub-pool B | 0.25 | 0.09 | 2.15 | 70.39 | 23.25 | 2.69 | 1.18 |
| Sub-pool C | 0.20 | 0.04 | 0.89 | 71.01 | 23.93 | 2.69 | 1.24 |
| Sub-pool D | 0.20 | 0.02 | 0.53 | 70.57 | 24.49 | 2.97 | 1.22 |
| Sub-pool E | 0.14 | 0.03 | 0.39 | 70.59 | 24.85 | 2.87 | 1.12 |
| Sub-pool F | 0.17 | 0.01 | 0.38 | 70.69 | 24.66 | 3.02 | 1.06 |
| Sub-pool G | 0.21 | 0.02 | 0.45 | 70.84 | 24.52 | 3.01 | 0.95 |
| Sub-pool H | 0.28 | 0.03 | 0.54 | 73.34 | 22.03 | 2.55 | 1.21 |
| Sub-pool I | 0.28 | 0.04 | 0.68 | 67.82 | 23.75 | 2.47 | 4.96 |

**TABLE S2** Glycan profile for feed fraction and sub-pools of mAb 12 eluted from Eshmuno CMX mixed-mode resin.

| Sample Description | Glycan Structure (Peak Area %) | | | | | | | | | | |
| --- | --- | --- | --- | --- | --- | --- | --- | --- | --- | --- | --- |
|  | G0F-N | G0 | G0F | Man5 | G1a | G1b | G1Fa | G1Fa | G2F | Total |  |
| Feed | 0.601 | 1.502 | 43.712 | 1.258 | 0.619 | 0.000 | 32.733 | 10.717 | 8.859 | 100.0 |  |
| A | 0.404 | 1.405 | 43.508 | 0.791 | 0.678 | 0.000 | 32.994 | 10.707 | 9.512 | 100.0 |  |
| B | 0.363 | 1.339 | 43.564 | 0.590 | 0.522 | 0.295 | 32.758 | 11.532 | 9.035 | 100.0 |  |
| C | 0.345 | 1.523 | 43.906 | 0.580 | 0.617 | 0.254 | 32.626 | 10.881 | 9.267 | 100.0 |  |
| D | 0.441 | 1.603 | 44.739 | 0.976 | 0.627 | 0.000 | 32.288 | 10.825 | 8.502 | 100.0 |  |
| E | 0.430 | 1.589 | 44.695 | 0.752 | 0.709 | 0.387 | 32.582 | 10.503 | 8.355 | 100.0 |  |
| F | 0.539 | 1.812 | 45.135 | 1.036 | 0.669 | 0.000 | 32.427 | 10.248 | 8.134 | 100.0 |  |
| G | 1.078 | 2.107 | 44.978 | 2.205 | 0.857 | 0.000 | 31.627 | 9.750 | 7.398 | 100.0 |  |
| H | 1.724 | 1.400 | 40.379 | 6.146 | 0.888 | 0.222 | 31.142 | 10.005 | 8.093 | 100.0 |  |
| I | 2.246 | 1.450 | 40.336 | 7.003 | 1.220 | 0.265 | 29.761 | 10.239 | 7.480 | 100.0 |  |
